# Complete Genome Sequence of *Mycobacterium massiliense* Clinical Strain Asan 50594, Belonging to the Type II Genotype

**DOI:** 10.1128/genomeA.00429-13

**Published:** 2013-07-05

**Authors:** Byoung-Jun Kim, Bo-Ram Kim, Seok-Hyun Hong, Seung-Hyeok Seok, Yoon-Hoh Kook, Bum-Joon Kim

**Affiliations:** Department of Microbiology and Immunology, Biomedical Sciences, Liver Research Institute, Institute of Endemic Diseases, Seoul National University Medical Research Center (SNUMRC), Seoul National University College of Medicine, Seoul, Republic of Korea

## Abstract

We report the complete genome sequence of the *Mycobacterium massiliense* clinical strain Asan 50594, which was grouped into the *M. massiliense* type II genotype, isolated from a Korean patient. This genome sequence will serve as a valuable reference for understanding the disparity in virulence and epidemiological traits between strains belonging to the *Mycobacterium abscessus* complex.

## GENOME ANNOUNCEMENT

Globally, *Mycobacterium abscessus* is the most commonly encountered pathogen among rapidly growing mycobacteria (RGM) (1). In South Korea, infection from *M. abscessus* is also the most prevalent RGM infection, second only to the *Mycobacterium avium* complex for nontuberculous mycobacterium (NTM) (2, 3). The recent application of multilocus sequencing has broadened our knowledge about the diversity of the strains in the *M. abscessus* complex (4, 5). Of these, *Mycobacterium massiliense* infections have gained importance since they cause soft tissue infection outbreaks (6) and pulmonary infections (7, 8). Recently, our group has introduced a novel *hsp65* genotype (type II) of *M. massiliense*, related to the rough colony morphotype, from Korean patients (9).

The aim of the present study is to introduce the complete genome sequence of *M. massiliense* clinical strain Asan 50594, belonging to the *M. massiliense* type II genotype (9). This strain was isolated from a patient with a pulmonary infection. The genome was sequenced by a standard shotgun strategy using GS FLX pyrosequencing technology. Sequencing analysis was performed at the National Instrumentation Center for Environmental Management (NICEM) (genome analysis unit) at Seoul National University. A total of 1,124,128 reads were generated, with an average read length of 342.7 bp, yielding 385,247,859 bp of total sequences. This represents ~77× coverage for the estimated 5.0-Mb chromosome size. The 61 contigs we obtained were compared for mapping to the whole-genome sequences of the reference strain using the BLASTz program (http://www.bx.psu.edu/miller_lab/). All the remaining gaps between contigs were completely filled by ~50-fold Solexa reads and PCR amplifications. The protein-coding genes (open reading frames [ORFs]) were predicted by Double ACT (http://www.hpa-bioinfotools.org.uk/pise/double_act.html) and the RAST server (10). The tRNAs and rRNAs were identified using tRNAscan-SE (11) and RNAmmer (12), respectively. Using the BLASTp program, each gene was identified by similarities and annotated. Finally, using Artemis (genome browser and annotation tool), the annotated ORFs were edited or corrected (13).

The *M. massiliense* type II (strain Asan 50594) genome has a circular DNA of 5,000,473 bp with two circular plasmids of 172,814 and 97,240 bp. It has a chromosomal size similar to those of *M. abscessus* CIP 104536^T^ (5,067,172 bp) and *M. massiliense* strain GO 06 (5,068,807 bp). It carries more protein-coding genes (4,958 ORFs) than *M. massiliense* strain GO 06 (4,313 ORFs), but the number of ORFs is similar to that for *M. abscessus* CIP 104536^T^ (4,920 ORFs). The *M. massiliense* type II genome includes 47 tRNA genes and one rRNA operon, comprising 5S, 16S, and 23S rRNA genes. The genome of *M. massiliense* type II and two plasmids have G+C contents of 64.22%, 64.18%, and 62.90%, respectively.

### Nucleotide sequence accession numbers.

The whole-genome sequences of *M. massiliense* type II strain have been deposited at GenBank under the accession no. CP004374 to CP004376.
